# Postharvest Losses Along the Small‐Scale Fish Value Chain in Bangladesh: Perceptions, Determinants and Implications for Food Security

**DOI:** 10.1002/fsn3.71809

**Published:** 2026-05-06

**Authors:** Md Jakiul Islam, Ripa Das, Tanni Sarkar, Shahir Chowdhury, Habiba Khan Ilham, Esrat Jahan, Abu Hayat Md. Saiful Islam

**Affiliations:** ^1^ Department of Fisheries Technology and Quality Control, Faculty of Fisheries Sylhet Agricultural University Sylhet Bangladesh; ^2^ Faculty of Fisheries Sylhet Agricultural University Sylhet Bangladesh; ^3^ Department of Agribusiness and Marketing Bangladesh Agricultural University Mymensingh Bangladesh; ^4^ Department of Agricultural Economics Bangladesh Agricultural University Mymensingh Bangladesh

**Keywords:** Bangladesh, determinants, fish, postharvest losses, regression analysis, value chain

## Abstract

Reducing postharvest losses (PHL) is a key pathway to achieving sustainable development goals, particularly food and nutrition security, in developing countries like Bangladesh. A slim body of literature assessing fish PHL indicates significant variation in estimates of loss across value chain actors and regions. We analyze a combination of questionnaire loss assessment method (QLAM) and sensory evaluations based on PHL from different value chain actors: farmers and traders, and perceived causes for losses by the VC actors, including consumers, based on a survey of farmers, traders, and consumers from northeast Bangladesh, one of the freshwater fish‐producing hubs in Bangladesh. PHL results indicate that average PHL, including physical, quality, and market losses, incurred about 10% and 12% at the farmer and trader levels, respectively. Causes of postharvest fish losses in the study area include unfavorable weather conditions, including high temperatures; lack of storage facilities; spoilage; poor fish handling practices; volatile demand and supply; long waiting times; communication gaps between actors; and inefficiencies at collection points. Ordinary least squares (OLS) regression and fractional probit regression results show that those who are younger, highly educated, more experienced, trained, and members of professional associations, have knowledge of cooling methods, sold their fish in short‐distance markets, and used better transport facilities incurred significantly less PHL. Therefore, to reduce fish PHL along the value chain and thereby ensure food security and nutrition, the government should invest in infrastructural facilities, including roads, markets, and storage for the small‐scale fisheries sector, and provide training on fish handling and PHL reduction strategies throughout the fish value chain in Bangladesh.

## Graphical abstract

Postharvest fish losses in Bangladesh remain substantial (about 10% for farmers and 12% for traders), driven by high temperatures, inadequate storage, poor handling practices, and market inefficiencies. Strengthening education and training, improving cooling practices, and enhancing transport and market access can significantly reduce these losses. Therefore, targeted investments in infrastructure, cold storage, and capacity building are essential to minimizing postharvest losses and improving food and nutrition security.

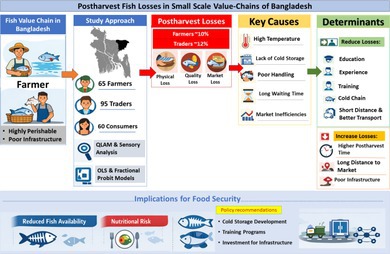

## 1 Introduction

Fish has long been a cheap, popular source of animal protein for most people. Fish contribute significantly to the animal protein supply of many communities in both the developed and developing worlds (Shamsuzzaman et al. 2020; Toufique and Belton 2014), and they are the primary source of animal protein for around 1 billion people worldwide (Belton et al. 2020; Naylor et al. 2000). Besides, fish act as a source of direct and indirect employment opportunities for rural communities (Theuerkauf et al. 2019). Fish are a vital aspect of food security and nutrition, especially for many impoverished people in developing nations. They account for 22% of total animal protein intake in low‐income food‐deficient countries (Belton et al. 2020; Subasinghe et al. 2009; Theuerkauf et al. 2019; Thilsted et al. 2016). Food loss is a worldwide topic in academia, policy‐making, and the fishery industry (Cattaneo et al. 2021; Fiore et al. 2019), with fish experiencing the highest losses among all commodities (Tesfay and Teferi 2017; FAO 2010; Buzby et al. 2009). Fish are perishable and hence prone to large postharvest losses if intervention measures are not implemented. Postharvest loss refers to the measurable qualitative and quantitative food loss that occurs along the supply chain, from harvest to consumption or other end uses (De Lucia and Assennato 1994; Hodges et al. 2011; Jha et al. 2015; Kaiya 2014; Kimiywe 2015). Fish postharvest losses are among the greatest of any commodity in the food production chain (Subasinghe et al. 2009; Thilsted et al. 2016). Despite being an important component of the livelihood structure, fish postharvest activities in the fisheries value chain have frequently received less attention in rural community development programs (Mustapha et al. 2013; Paul and Vogl 2013). However, because of its importance in the food basket, unique nutritional qualities, and superior production efficiency when compared to other agricultural food systems, fish need more attention in food policies than they now receive (FAO 2018; Thilsted et al. 2016). Fish and fish products are currently among the most traded food products on a global scale (FAO 2018). Significant advances in fish trading for both domestic and international markets are largely dependent on the quality of postharvest fisheries activities (Funge‐Smith and Bennett 2019; Theuerkauf et al. 2019). Global fish losses are estimated to be between 10 and 12 million tons per year, accounting for roughly 10% of total captured fisheries and aquaculture production (Ward and Signa 2014). As a result, combating climate change and hunger requires more than just increasing production and total food supply; it also necessitates improved food systems, a thorough understanding of local conditions and factors influencing product value chains, and a greater focus on the barriers impeding investment in improved postharvest handling practices, technologies, and policy (Chauhan et al. 2021; Edwards et al. 2019).

Fish is a major food item in Bangladesh, accounting for up to 60% of the national dietary animal protein (DoF 2019b). The country's current annual fish production is estimated at 4,610,000 tons from both capture and aquaculture fisheries. Bangladesh can increase its fish and fish product output. This is due to its vast freshwater, marine water, and land resources, along with abundant human capital (DoF 2019a). However, the country's fish supply and value chain face considerable postharvest losses. These losses are largely ignored (Ward and Signa 2014). During transportation and retail sales, fish are often kept without adequate cool chain management. Purposeful investment to avoid or eliminate postharvest food losses could reduce the supply–demand imbalance (Ward and Signa 2014; Mujuka et al. 2020; Yap 2013). Achieving these improvements requires producers in target locations to thoroughly understand postharvest operations and preservation procedures. In general, information on postharvest fish losses among various actors in Bangladesh's fishing sector remains unclear. The limited literature focuses only on aquaculture and capture fisheries, leaving postharvest losses unaccounted for. Most of the previous studies (Acharjee et al. 2021; Prodhan et al. 2022; Mandal et al. 2024; Acharjee et al. 2025; Mandal et al. 2025) used the questionnaire loss assessment method (QLAM). In contrast, this study used both the sensory evaluations proposed by Howgate (2011) and the QLAM method to better account for postharvest quantity and quality losses. Most of the earlier research did not comprehensively measure postharvest losses, especially quality losses. They considered only a few, mainly socio‐economic factors, responsible for losses in Bangladesh and elsewhere. For effective policy and planning, a comprehensive estimation and identification of a wide range of socio‐economic, policy, and infrastructural determinants are necessary.

Therefore, this study aims to address the following objectives: The specific objectives of this study are to: (i) quantify physical, quality, and market postharvest losses among fish farmers and traders (kg/100 kg); (ii) identify socio‐demographic, institutional, and infrastructural determinants of postharvest losses using OLS and fractional probit models; (iii) examine value chain actors' perceptions of loss causes and mitigation strategies; and (iv) assess the implications of postharvest losses for food security and policy design. To achieve these objectives, this study uses primary data collected from fish value chain actors from the northeastern part of Bangladesh, the greater Sylhet region, which is one of Bangladesh's major aquaculture‐producing hubs and is among the country's major fishery communities. Major haor systems in this area harbor the country's freshwater capture fishery as well as aquaculture production (DoF 2019a). Despite the strong potential for fish production in Sylhet (Mustafa et al. 2019), fish postharvest losses in the supply and value chain of greater Sylhet are still largely unexplored. Therefore, this study aimed to investigate postharvest fish losses and preservation practices in the northeastern part of Bangladesh.

## 2 Typical Small‐Scale Fish Value Chain in Bangladesh

**FIGURE 1:**
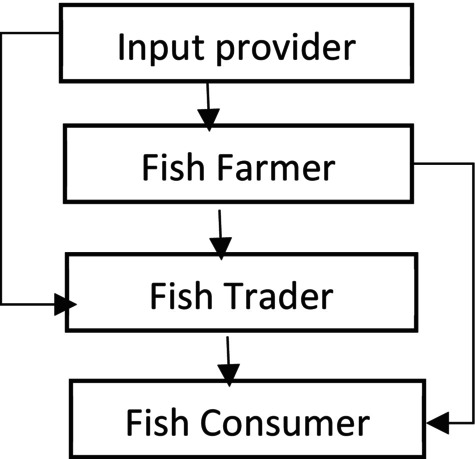
A typical fish value chain in Bangladesh.

Millions of people rely on small‐scale fisheries for a living, particularly in low‐income countries (Béné et al. 2010). A value chain analysis identifies opportunities to upgrade by enhancing quality and manufacturing processes, enabling greater value or product diversification (Rosales et al. 2017). The value chain describes a sequence of integrated economic activities and actors that bring a good or service to the market, adding incremental value to the product at each node of the chain (Porter 1985; Sturgeon 2001). Value can be added, for example, through processing or grading products based on different quality attributes (Bjorndal et al. 2014). A study conducted by Acharjee et al. (2023) revealed that the fish transferring process from producers to consumers involves different actors, which include *Bepari*, rural *Paikar*, *Aratdar*, urban *Paikar*, and retailer, where fish farmers drive transformation by converting input into output, adding value through production, whereas managing farming operations, making timely decisions, and securing financing. Small‐scale fisheries value chains encompass various actors and activities, including gear production, fish capture, sorting, cleaning, processing, transportation, marketing, selling, and the consumption of fresh fish and fish products (FAO 2020a, 2020b; Islam 2016; Islam and Hasan 2020). Numerous tasks within the value chains of small‐scale fisheries are performed manually, frequently employing inexpensive tools for the handling and processing of fish. Limited postharvest infrastructure and facilities, a lack of fish product quality control and certification systems, a paucity of processed fish, a lack of training facilities, and poor fisheries: among the obstacles to sustainable management of small‐scale fisheries value chains are weak fisheries governance systems (Jacinto and Pomeroy 2011; Kimani et al. 2020; Rosales et al. 2017; Galappaththi et al. 2021). A typical small‐scale fish value chain is shown in Figure 1, where four categories of actors are involved in fish value chain activities. Within these four categories, there are multiple actors, including input providers such as fingerlings, feed, labor, and other inputs like ice; transport providers are also involved, and all these actors supply various inputs to fish farmers as well as to fish traders. Fish farmers in Bangladesh also characterize different categories, including small, medium, and large in terms of farm size, extensive, semi‐intensive, and intensive in terms of stocking density, and monoculture and polyculture in terms of species combination stocked. Similarly, fish traders also have different categories like wholesalers and retailers in terms of volume of operation, also locally known as *Bepari*, *faria, Paikar*, and *Aratdar*. Postharvest losses occur at multiple stages along the value chain. Various studies have examined the determinants of value chain participation among different actors along the chain (Islam 2021), and such participation has been shown to positively affect the welfare of value chain actors (Islam 2024).

## 3 Materials and Methods

### 3.1 Selection of the Study Areas

**FIGURE 2:**
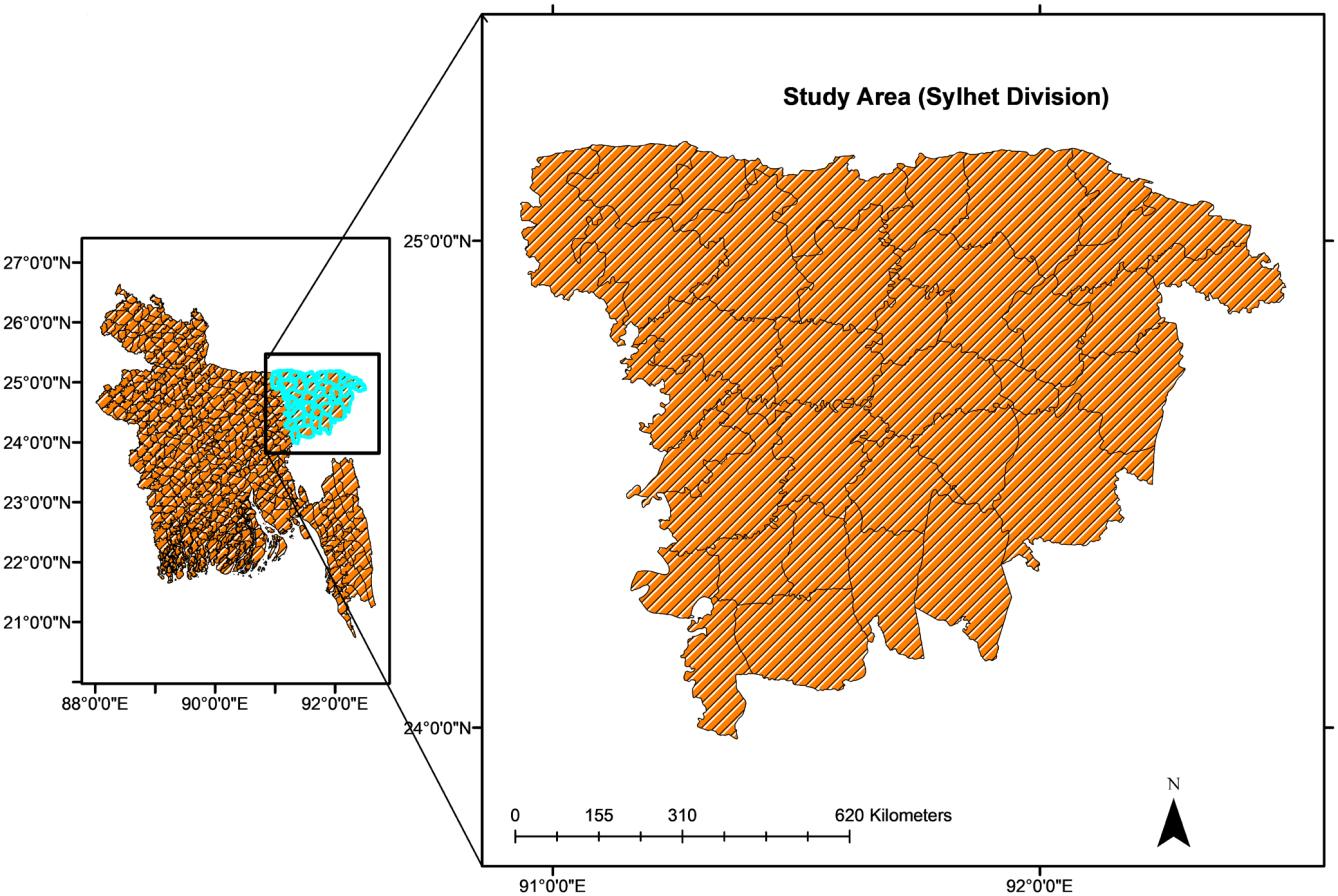
Map showing study area.

The study was conducted in the northeastern part (Greater Sylhet area) of Bangladesh. It focused on Sunamgonj, Habigonj, and Sylhet districts (Figure 2), where fish production and capture are higher. Additionally, fish from outside the Sylhet region were also considered. For each of the selected districts, at least three sub‐districts (Upazilas) were chosen from each district for data collection to comprehend the local knowledge, attitudes, and practices of fish stakeholders including farmers, sellers, and consumers. Furthermore, the major fish supply chain was traced to collect fish samples for laboratory analyses aimed at understanding postharvest losses. Three representative fish species—tilapia ( *Oreochromis niloticus* ), pangas ( *Pangasius hypophthalmus* ), and pabda (*Ompok bimaculatus*)—were selected for this study due to their status as the most consumed fish species in Greater Sylhet. Among these, pangas emerged as the dominant species, constituting approximately 23.2% of cultured fish species. Other species, such as tilapia, contribute 12.58% and 16.64% to total aquaculture production, respectively. Pabda, being the most popular fish among small indigenous species in the study area, holds a significant presence (DoF 2023).

### 3.2 Data Collection

Before data collection began, coordination was established with the Department of Fisheries (DoF) to obtain demographic information about fishers and aquaculture producers in the district. During pre‐testing, insights into the best times for interviews and information collection about postharvest activities were also sought. Three distinct sets of questionnaires were designed for farmers, sellers, and consumers. These covered key aspects such as demographics, fish catches, losses, and preservation practices. The questionnaires were rigorously pre‐tested before data collection started. Necessary refinements were made to improve their effectiveness. The primary focus of the questionnaires included inquiries into: (1) the extent of postharvest fish losses in the study area; (2) the strategies used to mitigate such losses; (3) the preservation practices adopted by stakeholders; (4) the effectiveness of these preservation practices; (5) adherence to fish product safety and quality control guidelines; and (6) major challenges causing postharvest fish losses in the district. Field visits with respondents were conducted between March and August 2023. These visits aligned with guidance from the DoF. The respondent group included 65 farmers, 95 sellers (both retailers and wholesalers), and 60 randomly selected consumers from the study areas. Data were collected through semi‐structured survey questionnaires and face‐to‐face interviews. Additionally, insights into overall fisheries activities were gathered through direct observation and discussions with farmers, sellers, and consumers along the fish value chain. Before each interview, researchers provided a comprehensive explanation of the research objectives to every respondent. This ensured clarity and transparency throughout the process.

### 3.3 Measurement of Postharvest Losses

**FIGURE 3:**
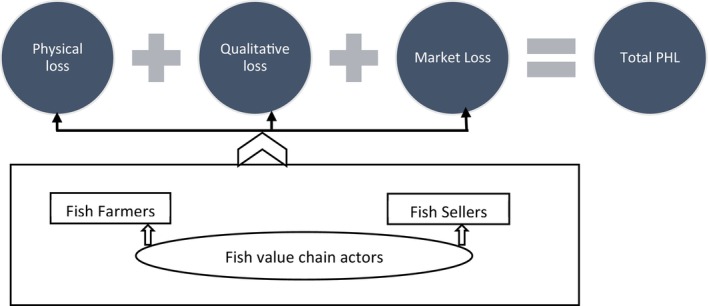
Conceptual framework of fish PHL among the value chain actors.

The term postharvest loss refers to “a measurable quantitative and qualitative loss in a given product… [and] restriction in the use of the product… [whereby] the sum of losses in quantity and quality of the products inevitably means losses of food and money” (Lucia and Assennato 1994; Grolleaud 2002). Reliable measurement of postharvest food losses is crucial for sound policy making towards postharvest losses reduction (Bellemare et al. 2017). This study estimates physical, quality, and market (economic) losses, using the framework in Figure 3. Total postharvest losses (PHL) were estimated by adding three types of losses. Physical losses were defined as the actual reduction in quantity or weight of fish due to spoilage, breakage, discards, and leakage during handling and transportation. These losses were quantified by recording the initial weight of fish at the retailers and comparing it with the final weight observed at the retail point. The percentage loss was calculated accordingly. Direct observations and interviews with traders and retailers validated the causes of these losses. Most existing studies (Acharjee et al. 2021; Prodhan et al. 2022; Mandal et al. 2024; Acharjee et al. 2025; Mandal et al. 2025) rely on the questionnaire loss assessment method (QLAM). In contrast, this study assessed quality losses through sensory evaluations, following the methods proposed by Howgate (2011). Organoleptic properties such as color, odor, texture, and general appearance were scored using a 5‐point hedonic scale, with trained evaluators following FAO guidelines. Sensory quality assessment used a structured scoring system adapted from FAO postharvest loss assessment guidelines (FAO 1999). Fresh fish were judged on appearance (skin brightness, gill color), odor, texture, and eye clarity. Each attribute was scored on a scale from 1 (excellent freshness) to 5 (unacceptable spoilage). Fish with an average sensory score above 3 were classified as quality deteriorated. Quality loss (kg/100 kg) was calculated as the proportion of fish downgraded due to sensory deterioration, multiplied by the total quantity handled. The formula used was: Quality Loss (kg/100 kg) = (Quantity downgraded due to sensory deterioration/Total quantity handled) × 100. This approach translates sensory deterioration into quantitative loss estimates while maintaining consistency with the QLAM framework. Results were interpreted with reference to established food safety thresholds such as those set by the European Union and Codex Alimentarius. Market losses helped understand the economic impact of quality degradation and unsold fish. These were estimated by comparing the expected market value of fresh fish to the actual price received for downgraded or deteriorated fish, based on daily price monitoring and stakeholder responses. Physical and market losses were calculated using the QLAM method and its related formula (Diei‐Ouadi and Mgawe 2011; Ward 1997; Prodhan et al. 2022; Rasheduzzaman et al. 2024). OLS coefficients represent the absolute change in postharvest loss (kg/100 kg) associated with a one‐unit change in the explanatory variable, holding other factors constant. Marginal effects from the fractional probit model show the change in the expected proportion of loss for a one‐unit change in the explanatory variable. Due to the observational nature of the data, results are interpreted as associations, not causal relationships. To enhance the reliability of the postharvest loss estimate, the inquiry was restricted to the most recent farmer harvest and the most recent sale by sellers prior to the survey. Structured interviews were conducted across the value chain. These interviews gathered insights into loss perception, handling behavior, infrastructure use (such as ice and cold storage), and constraints contributing to postharvest inefficiencies.

### 3.4 Empirical Research Method: Ordinary Least Squares Regression and Fraction Probit Models

Quantitative data collected from field surveys and laboratory tests were analyzed using STATA. Data for the study were analyzed using descriptive statistics and inferential statistics such as mean, minimum, maximum, standard deviation, frequency, and percentage to summarize socio‐demographic aspects of the value chain actors. To begin with, considering the dependent variable is continuous (quantity of fish loss for this study), at first, the multiple linear regression model using the ordinary least squares (OLS) method was applied as a part of inferential statistics to find out the determinants of fish postharvest losses at both the farmers' and traders' levels. The multiple regression model is simple, easy to interpret, and practically applicable (Greene 2000). Numerous studies on determinants of postharvest loss assessment have also used a multiple linear regression model using the OLS method (Acharjee et al. 2021; Prodhan et al. 2022; Mandal et al. 2024; Acharjee et al. 2025; Mandal et al. 2025). To test the correlation of the independent variables used in the model, a multicollinearity test by applying the variance inflation factor (VIF) was conducted. VIF ranged between 1 and 3.5 (and mean 3.5 and 4.5 at the farmers' and sellers' levels, respectively), indicating that there was no significant multicollinearity between the independent variables (Table A1). The empirical form of the regression model for the determinants of postharvest losses at the farmers' level is specified as follows:

$$Y_{i}=\beta_{0}+\beta_{i}\;X_{i}+\mu_{i}$$

where *Y*_*i*_ refers to the postharvest fish losses for each fish farmer or seller (kg/100 kg). *X*_*i*_ (*i* = 1–16) are the explanatory variables. *β*_0_ is the intercept, *β*_*i*_ is the coefficient of explanatory variables, and *μ*_*i*_ is the disturbance term. The explanatory variables of the models were selected based on a review of relevant literature and field experience (see Table 1). If all value chain actors incur postharvest loss, the OLS estimation method is ideal. However, not every value chain actor may incur postharvest losses. Some actors might not have any losses for various reasons. When using OLS regression and including only value chain actors with losses, sample selectivity bias may occur. In this study, we address this issue. We also seek to check the robustness of the results and account for the fractional dependent variable: the proportion of fish losses per 100 kg. To do so, we use the method of Papke and Wooldridge (1996), applying a fractional probit model to estimate the factors affecting fish postharvest losses. Literature shows that for such a dependent variable, OLS, censored regression (Tobit), or the transformed logistic normal model may be inefficient due to heteroskedastic error distributions (Papke and Wooldridge 1996; Gallani and Krishnan 2017). The fractional probit model allows the dependent variable to be continuous within bounded regions. The following functional form specifies the expected value of postharvest *Y*_*i*_ for the *i*th household, given explanatory variables *X*_*i*_:

(1) $$E\left(Y_{i}/X_{i}\right)=Z\;\left({\mathrm{BX}}_{i}\right)$$

where *Y*_*i*_ denotes the proportional quantity of fish postharvest loss (kg/100 kg) of the *i*th household, *X*_*i*_ is a vector of explanatory variables (Table 1), and *β*_*i*_ represents a vector of coefficients to be estimated. *Z*(·) is the normal cumulative distribution function, and *E* is the expectation operator. To analyze these relationships, the fractional probit model is specified in Equation (1). This model is non‐linear and estimated using the Quasi‐Maximum Likelihood Estimation (QMLE) method, as outlined in Wooldridge (2016). Compared to either OLS or Tobit, QMLE is asymptotically efficient and consistent. To account for potential heteroscedasticity, robust standard errors are estimated, and marginal effects are also calculated in STATA.

**TABLE 1:** Descriptive statistics of variables used in regression analysis.

Variables	Description	Farmers (*N* = 65)		Sellers (*N* = 95)		Literature
		Mean (frequency)	SD (%)	Mean (frequency)	SD (%)	
Age category	Age category of farmers 16–25 = 1, 26–35 = 2, 36–45 = 3, 46–55 = 4, 56–65+ = 5	3.69	0.92	2.79	1.02	Adelaja et al. (2018), Tunsisa et al. (2024), Mramba and Mkude (2022) and Agbebi et al. (2021)
	16–25	0	0	8	8.42	
	26–35	6	9.23%	31	32.6	
	36–45	22	33.85%	35	36	
	46–55	23	35.38%	15	15	
	56–65+	14	21.54%	6	6	
Education	Education category of farmers Primary = 1, High school = 2, SSC = 3, HSC = 4, Diploma = 5, Bachelor = 6, Masters = 7	2.25	1.51	1.89	1.08	Adelaja et al. (2018), Tunsisa et al. (2024), Mramba and Mkude (2022) and Agbebi et al. (2021)
	Primary	24	36.92%	45	47.37	
	High school	20	30.77%	30	31.5	
	SSC	15	23.08%	5	5.2	
	HSC	1	1.54%	15	15.8	
	Diploma	0	0	0	0	
	Bachelor	2	3.08%	0	0	
	Masters	3	4.62%	0	0	
Occupation	Occupation of farmers/sellers Fish farming = 1, Business = 2, Service = 3	1.29	0.58	1.45	0.66	Mandal et al. (2025) and Obar et al. (2025)
	Fish farming	50	76.92	61	64.21	
	Business	11	16.92	25	26.32	
	Services	4	6.15	9	9.47	
Engagement with fish farming/selling	Years of engagement with fish cultivation 1–5 = 1, 6–10 = 2, 11+ = 3	2.09	0.76	2.04	0.87	Mandal et al. (2025), Adelaja et al. (2018), Tunsisa et al. (2024), Agbebi et al. (2021) and Obar et al. (2025)
	1–5	16	24.62	34	35.79	
	6–10	27	41.54	23	24.21	
	11+	22	33.85	38	40	
Received Training	Training on fish cultivation/preservation Yes = 1, No = 0	0.35	0.48	0.07	0.26	Obar et al. (2025)
	Yes	23	35.38%	7	7.37	
	No	42	64.62%	88	92.63	
Engagement with association	Engagement with fish farm/seller association Yes = 1, No = 0	0.29	0.46	0.41	0.49	Tunsisa et al. (2024)
	Yes	19	29.23	39	41.05	
	No	46	70.77	56	58.95	
Contact with fish personnel	Contact with fish personnel Yes = 1, No = 0	0.23	0.42	0	0	Tunsisa et al. (2024)
	Yes	15	23.08	0	0	
	No	50	76.92	0	0	
Positive Perception of cooling method	Positive perception of cooling method to keep fish in good quality Yes = 1, Otherwise = 0	0.62	0.49	0.79	0.41	Mandal et al. (2025) and Obar et al. (2025)
	Yes	40	61.54	75	78.98	
	Otherwise	25	38.46	20	21	
Perception about quality of iced fish	Positive perception about iced fish quality Yes = 1, Otherwise = 0	0.54	0.50	0.80	0.40	
	Yes	35	46.15	76	80	
	Otherwise	30	53.85	19	20	
Place of fish sale	Place of fish sale Farm gate = 1, Neighbor = 2, Village market = 3, Upazila market = 4, Dist market = 5, Local dipo = 6, Main dipo = 7	3.05	1.66	2.22	0.66	
	Farm gate	22	33.85	0	0	
	Neighbor	3	4.62	0	0	
	Village market	8	12.31	12	12.63	
	Upazila market	14	21.54	50	52.68	
	, Dist market	18	27.69	33	34.74	
	Local dipo					
Mode of transportation	Mode of transportation Rickshaw & van = 1, Refrigerated van = 2, Truck & pickup = 3, Bus & others = 4	3.03	0.71	2.97	0.94	Wibowo et al. (2017)
	Rickshaw & van	4	6.15	13	13.68	
	Refrigerated van	3	4.5	4	4.21	
	Truck & pickup	45	69	51	53.68	
	Bus & others	13	20	27	28.42	
Distance to fish market	Distance to fish market from home	5.08	3.01	10.95	11.65	Tunsisa et al. (2024) and Agbebi et al. (2021)
Transportation facility	Type of transportation facility Good = 1, Not good = 0	0.74	0.44	1.93	0.34	
	Good	48	73.85	67	70.53	
	Not good	17	26.15	28	29.47	
Infrastructure facility	Type of infrastructure facility Good = 1, Not good = 0	0.71	0.46	0.71	0.46	Agbebi et al. (2021) and Acharjee et al. (2025)
	Good	46	70.77	53	56	
	Not good	19	29.23	42	44	
Use preservatives	Use preservatives Yes = 1, No = 0	0	0	0.28	0.45	
	Yes	0	0	27	29	
	No	0	0	68	71	
Cooling facilities	Have cooling facilities Yes = 1, No = 0	0	0	0.33	0.47	
	Yes	0	0	31	33	
	No	0	0	64	67	

## 4 Results and Discussion

### 4.1 Descriptive Statistics

Table 1 presents descriptive statistics of fish farmers and sellers in the study area. Most fish farmers (35.38%) are aged 46–55, and 34% are 36–45 years, similar to findings by Acharjee et al. (2025), Adelaja et al. (2018), Kyangwa and Odongkara (2005), and Mungai (2014). Fish farmers are active regardless of age. Conversely, 36% of sellers are 36–45 years old, and 32% are 26–32, indicating they are generally younger. About 37% of farmers and 47% of sellers have primary education; 31% of both attended high school, with 23% of farmers passing the SSC exam. Omwega et al. (2006), Mungai (2014), and Tesfay and Teferi (2017) reported similar results. Low education may affect acceptance of new handling practices (Onemolease and Oriakhi 2011). Adelaja et al. (2018) also found that most fishermen had secondary education, which may make them more open to new practices. Seventy‐seven percent of respondents listed fish farming as their main occupation; 17% combined it with other businesses. Among sellers, 64% are also fish farmers, and 26% have other business activities, showing most rely mainly on fishing income, consistent with Adelaja et al. (2018). The majority of fish farmers have 6–10 years (41.54%) or more than 10 years (34%) of experience, whereas 35% of sellers have 1–5 years. Thus, fish farmers have more experience than sellers. Most fish farmers (64%) and sellers (92%) have no training in fish cultivation, suggesting limited awareness of modern technology or proper fish handling.

Regarding fish associations, 71% of farmers and 59% of sellers are not members, whereas Adelaja et al. (2018) observed higher membership rates among farmers. Most fish farmers (77%) have contact with fish personnel and thus have access to expert information. A majority of farmers (61%) and sellers (79%) know about cooling methods, with 54% of farmers and 80% of sellers using icing, whereas others use chilling or freezing. The survey found 33% of fish farmers sell at the farm gate and 27% at district markets, possibly for better prices. Trucks and pickups are used for transporting fish by 69% of farmers and 54% of sellers. The average market distance is 5 and 10 km for farmers and sellers, respectively. Around 74% of farmers and 70% of sellers reported good transport, and 70% of farmers and 56% of sellers had good infrastructure. Most sellers (71%) used no preservatives, and 67% lacked cooling facilities.

### 4.2 Fish VC Actors' Perception and Attitudes on Preservation Practices

Table 2 shows the perception and attitude of different value chain actors, including farmers, sellers, and consumers, towards fish preservation practices. The majority of farmers (61%), sellers (81%), and consumers (76%) agreed that using cooling methods keeps fish in good quality compared to no cooling at all. Meanwhile, 58% of farmers, 89% of sellers, and 72% of consumers were aware of different cooling methods such as icing, chilling, and freezing. Regarding iced fish quality, 58% of farmers, 79% of sellers, and 58% of consumers agreed it was better in the study area. Most farmers (55%), sellers (72%), and consumers (71%) preferred iced fish. However, 38% of farmers, 17% of sellers, and 26% of consumers reported very poor preferences for iced fish. Almost 96% of sellers and farmers, and 90% of consumers, agreed that raw fish quality should be maintained and kept as fresh as possible.

**TABLE 2:** Perception and attitude towards fish preservation practice.

VC actors' perception	Farmers (*N* = 65)	%	Sellers (*N* = 95)	%	Consumer (*N* = 60)	%
Cooling method keep fish good (= 1 if yes)	40	61.54	77	81.05	47	75.81
Have an idea on fish cooling method (= 1 if yes)	38	58.46	85	89.47	45	72.58
Iced fish is good quality (= 1 if yes)	35	53.85	75	78.94	36	58.06
*Preferences for iced fish*						
Excellent	4	6.15	5	5.26	1	1.67
Good	36	55.38	69	72.63	43	71.67
Poor	25	38.46	17	17.89	16	26.67
Quality of raw fish should be as fresh as possible (= 1 if yes)	63	96.92	92	96.84	56	90.32

*Note:* The cumulative sum of the percentage is not always 100% since multiple counts are accounted for.

Different preservation practices used by value chain actors are presented in Table 3. The result shows that the majority of farmers (46%) and sellers (73%) used the icing technique while 64% of consumers used the freezing technique as a cooling method, which is that refrigerators are easily accessible for consumers while the icing method is more convenient for farmers and sellers. Only 3% of farmers and 7% of sellers used ice that was of excellent quality. Different icing methods were used by the value chain actors; among them, the wooden box was the most used method by the fish farmers (38.46%), followed by other methods such as foam and open ice. Although sellers mostly used open ice and the foaming method, consumers' most preferred method was using a plastic box. The value chain actors also used various types of ice, such as block, crushed, and flake ice, and among them, crushed ice was the most used type by farmers (46.15%), sellers (57.89%), and consumers (64.52%).

**TABLE 3:** Preservation practices used by fish VC actors.

Preservation practices	Farmers (*N* = 65)	%	Sellers (*N* = 95)	%	Consumer (*N* = 60)	%
*Use of the cooling method*						
Icing	30	46.15	70	73.68	11	17.74
Chilling	7	10.77	17	17.89	6	9.68
Freezing	1	1.54	1	1.05	40	64.52
Others	27	41.54	7	7.37	5	8.04
*Quality of ice used*						
Excellent	2	3.08	7	7.37	0	0
Very good	12	18.46	31	32.63	4	6.45
Good	23	35.38	47	49.47	32	51.61
Poor	1	1.54	4	4.21	26	41.94
*Icing method used*						
Plastic box	16	24.62	17	17.89	28	45.16
Wooden box	25	38.46	36	37.89	14	22.58
Others (e.g., foam, open ice)	24	36.92	42	44.21	20	32.26
*Types of ice*						
Block	14	21.54	14	14.74	9	14.52
Crushed	30	46.15	55	57.89	40	64.52
Flake	21	32.31	26	27.37	13	20.97

*Note:* The cumulative sum of the percentage is not always 100% since multiple counts are accounted for.

### 4.3 Postharvest Losses at the Farmers and Traders Level

Table 4 shows different types of postharvest losses at both farmers' and traders' levels. Total postharvest loss was 10 kg on average per 100 kg at the farmers' level, among which 3.5 kg was physical loss, 3.21 kg was quality loss, which could be due to improper cooling and handling technique, and 3.49 kg was market loss. On the contrary, at the trader's level, the total postharvest loss was 12.31 kg, which is more than that of farmers, where 3.31 kg was physical loss, and quality loss, as well as market loss was 4.52 and 4.48 kg, respectively.

**TABLE 4:** Postharvest losses at farmers and traders level (kg/100 kg).

Variables	Farmers (*N* = 65)				Sellers (*N* = 95)			
	Mean	SD	Min	Max	Mean	SD	Min	Max
Physical loss (kg/100 kg)	3.51	1.86	0	10	3.31	1.99	0	12
Quality loss (kg/100 kg)	3.21	2.30	0	9	4.52	2.68	0	15
Market loss (kg/100 kg)	3.49	2.09	0	10	4.48	2.95	0	15
Total postharvest loss (kg/100 kg)	10.21	4.97	3	20	12.31	5.28	3	36

### 4.4 How the Farmers and Traders Deal With Unsold and Spoiled Fish

During the value chain of fish, farmers and traders face the problem of fish spoilage and unsold fish, and they have some dealing mechanisms for this problem. In the survey area, it was found that the most common mechanism was discarding the fish for both the farmer (40%) and seller (21.05%), whereas 29.23% of farmers and 21.05% of sellers sold their product at a low price, as it would help them recover some cost. There were some other mechanisms for dealing with such problems, which were distributing them among neighbors and the poor (not adopted by the sellers), drying them so that they can prevent spoilage, and selling them as a transformed product, keeping some in ice, and also burying them far from the farm (Table 5).

**TABLE 5:** Farmers and traders dealing mechanisms of unsold and spoiled fish.

Dealing with unsold and spoiled fish	Farmers (*N* = 65)	%	Sellers (*N* = 95)	%
Discarded	26	40.0	20	21.05
Distribute among neighbors and poor	9	13.85	5	5.26
Own consumption	7	10.77	6	6.31
Sell at lower price	19	29.23	20	21.05
Keep in ice	1	1.5	11	11.58
Buried far from farm	1	1.5	0	0
Make dried fish	16	24.62	15	15.79
No fish remains unsold	3	4.61	5	5.26

*Note:* Cumulative sum of the percentage is not always 100% since multiple counts are accounted.

### 4.5 Perceived Causes of Fish Postharvest Losses

Value chain actors of the study area were asked to respond about their perspective regarding the causes of fish postharvest losses, where they identified 8 probable causes; among them, high temperature or adverse weather conditions were ranked first by both the sellers and farmers, whereas poor fish handling practices ranked first based on the consumers' perspective. Lack of storage facility and spoilage of fish were the 2nd and 3rd ranked causes responded by both seller and farmer. Other causes were unexpected demand and supply, long waiting time or lack of immediate actors, lack of effective communication, and inefficiencies at the collection points (Table 6).

**TABLE 6:** VC actors perceived causes of fish postharvest losses.

Reasons for fish postharvest losses	Rank		
	Farmers (*N* = 65)	Sellers (*N* = 95)	Consumers (*N* = 60)
High temperature/weather condition	1	1	2
Lack of storage facilities	2	2	3
Spoilage	3	3	4
Fish handling practices	4	4	1
Unexpected demand and supply	5	5	7
Lack of immediate actors/long waiting time	6	6	6
Lack of effective communication	7	7	8
Inefficiencies at collection points	8	8	5

### 4.6 Determinants of Postharvest Losses at Farmers and Traders Level

Table 7 shows the results of OLS and fraction probit estimation of different factors of postharvest losses at the farmers' level. The *F*‐statistics estimated were 18.87 and statistically significant at 5%, whereas the *R*‐squared value of the model was 0.93, which implies that 93% variation in loss incurred at the farmers' level is explained by the explanatory variables. The results of OLS estimation show that farmers aged between 46 and 55 and 56 and 65+, occupation of farmer as a service holder, fish selling at the upazilla market and district market, and distance of village market were positive determinants of fish postharvest loss. On the other hand, bachelor's and master's degree holding fish farmers, farmers' years of engagement in fish selling and fishing, training on fish preservation, fish farmers' contact with fish seller association, fish farmers' perception about cooling method, fish selling at neighborhood, and transportation facility in farmers' location were significant negative determinants of fish postharvest loss.

**TABLE 7:** Determinants of postharvest losses at farmer's level: Regression results.

Variables	OLS regression		Fraction probit regression			
	Total loss		Fraction loss		Marginal effects	
	Coef.	Robust SE	Coef.	Robust SE	d*y*/d*x*	Robust SE
*Age category*						
36–45	1.24	1.00	0.08*	0.05	0.01*	0.01
46–55	2.43**	0.96	0.14***	0.05	0.02***	0.01
56–65+	3.62***	1.05	0.19***	0.05	0.03***	0.01
*Education category*						
High school	−0.33	0.74	−0.01	0.03	0.00	0.01
SSC	0.85	0.63	0.05*	0.03	0.01*	0.01
HSC	1.44	1.29	0.15***	0.06	0.03**	0.01
Bachelor	−5.13***	1.63	−0.27***	0.07	−0.04***	0.01
Master	−2.88**	1.39	−0.22***	0.07	−0.03***	0.01
*Occupation*						
Business	1.22	1.06	0.04	0.04	0.01	0.01
Service holder	2.41**	0.95	0.07*	0.04	0.01*	0.01
*Years of engagement in fish farming (Base = 1–5)*						
6–10	−1.23*	0.65	−0.09***	0.03	−0.02***	0.01
11+	−1.51*	0.80	−0.09***	0.03	−0.02***	0.01
Training on fish farming	−1.41**	0.69	−0.09***	0.03	−0.02***	0.01
Engagement with fish farmers association	−1.57*	0.83	−0.09**	0.04	−0.02**	0.01
Contact with fish personal	0.82	0.87	0.05	0.04	0.01	0.01
Perception about Cooling method	−1.47**	0.71	−0.09***	0.03	−0.02***	0.00
Perception about frozen fish	−1.10	0.83	−0.08**	0.04	−0.01**	0.01
*Places of fish sale*						
Neighbors	−2.84***	0.95	−0.15***	0.05	−0.02***	0.01
Village market	0.50	1.04	0.01	0.06	0.00	0.01
Upazilla market	1.72**	0.82	0.12***	0.04	0.02***	0.01
Dist. market	1.74**	0.79	0.12***	0.04	0.02***	0.01
*Mode of transportation*						
Refrigerated van	−1.24	1.61	−0.12	0.10	−0.02	0.02
Truck or pickup	−1.18	0.94	−0.09*	0.05	−0.02*	0.01
Bus and others	0.14	1.33	0.00	0.07	0.00	0.01
Distance of village market	0.19**	0.10	0.01**	0.00	0.00**	0.00
Transportation facility in farmers location	−1.95***	0.67	−0.09***	0.03	−0.02***	0.01
Infrastructure facility in farmers location	−0.78	0.83	−0.04	0.04	−0.01	0.01
Constant	12.33***	2.24	−1.16***	0.11		
*F* (26, 37)/Wald *χ* ^2^ (27)	18.87***			1275.18***		
*R*‐squared/pseudo *R* ^2^	0.93			0.04		

*Note:* ∗, ∗∗, ∗∗∗ indicate significance at 10%, 5%, and 1% level, respectively.

The result of fraction probit is used as the amount of postharvest loss is a fractional variable which could not be considered fully by the above model and it shows all the variables have a significant relationship with fish losses after harvesting except high school education category, business persons, contact with fishing personnel, fish selling at village market, transporting these fishes using refrigerated van, and infrastructure facility in farmers' location. Age categories show a positive and significant relationship, which means the higher the age, the higher the chances of postharvest losses.

The results of the OLS model indicate that an increase in a farmer's age by 1 year leads to a rise in postharvest losses by 2.43% for those aged 46 to 55 years and by 3.62% for those aged 56 years and above, compared to the younger age group. Similarly, the marginal effect of the fractional probit model shows that a 1% increase in age results in a higher proportion of fish losses, with losses increasing by 0.01 for farmers aged 36 to 45 years, 0.02 for those aged 46 to 55 years, and 0.03 for those aged 56 years and above. This suggests that younger farmers are more familiar with modern fishing techniques and handling practices, leading to lower income losses than their older counterparts. Younger farmers are less risk‐averse than the older group, and they easily adopt new ideas on fish harvesting and marketing and are more flexible in the decision‐making process regarding new and modern technologies, which may result in lower postharvest loss (Langyintuo and Mungoma 2008; Malit et al. 2022).

Farmers with higher levels of education experience less fish loss after harvesting. The results indicate that farmers with a bachelor's degree face a 5.13% lower income loss, whereas those with a master's degree experience a 2.88% reduction compared to farmers with lower educational backgrounds. The marginal effect shows that a 1% age increase in education results in decreased income loss by 0.04 and 0.03 for bachelor's and master's degree holding farmers, respectively. Acharjee et al. (2021) and Chen et al. (2018) also found a negative association between higher education and postharvest fish loss. Educated fish farmers are more concerned about the quantity of fish loss due to poor harvesting and handling techniques and the consequences of this loss on income, and they are well aware of the use of advanced technology, enabling them to make informed decisions on fish postharvest losses, which was also found in a number of studies (Abiodun and Cookey 2023; Angermayr et al. 2023; Iruo et al. 2018; Kitinoja 2016; Ward and Signa 2014; Getu et al. 2015; and Abbas et al. 2024).

Service holder fish farmers incurred 2.41% more postharvest losses than those with no other alternatives except fish farming. And their proportion of income loss was 1% higher than that of those with only fish farming, which could be because of their inadequate time spent on fish farming. A study conducted by Mandal et al. (2024) found that fishermen who make fishing their only source of income are more likely to use superior handling and preservation practices, which lowers losses and increases profits. On the other side, those with other livelihoods might not put the best fish handling procedures first, which could result in more losses and lower revenue.

Farmers' years of experience in fish farming had a significant negative impact on fish postharvest losses. Farmers with 6–10 years of experience incurred 1.23% less postharvest loss, whereas those with more than 11 years of experience experienced 1.51% less loss compared to those with 1–5 years of experience. Additionally, the proportion of income loss due to postharvest losses was reduced by 0.02 for farmers with 6–10 years and more than 11 years of experience in fish farming. This finding suggests that greater experience leads to lower postharvest losses, aligning with the results of Agbebi et al. (2021). Fishermen with more years of experience employ better handling practices and preservation techniques, which result in reduced fish postharvest loss (Ejeta et al. 2024; Maulu et al. 2020).

Farmers with training on fish preservation had a significant negative influence on the proportion of income loss due to fish loss after harvesting. For a 1% increase in training, those farmers who had fish preservation training were more likely to reduce the proportion of income loss by 0.02 than those who had no such training. The study aligns with the findings of Ejeta et al. (2024), which indicate that inadequate training leads to more fish spoilage. Adelaja et al. (2018) and Acharjee et al. (2021) concluded that training facilities could significantly contribute to reduced postharvest loss. Training helps fishers to be aware of their limitations and learn skills that make them more effective at fishing (Purcell et al. 2018; Yanfika et al. 2019). Farmers' engagement with fish seller associations significantly influences postharvest fish loss. Farmers who are members of such associations experience a lower income loss of 0.02 due to reduced postharvest losses compared to those without association involvement. Fishers' participation with such associations gives them access to more information, resources, training, and networks, which leads to reduced postharvest losses and better economic outcomes (Abelti and Teka 2024; Ejeta et al. 2024).

Farmers' perception of cooling methods and frozen fish significantly influenced postharvest fish losses. Farmers with knowledge of different cooling methods experienced a lower proportion of income loss of 0.02 compared to those without such knowledge. Conversely, farmers with knowledge of frozen fish incurred a higher income by 0.01 compared to those without any perception of frozen fish. The use of ice and other cooling methods could significantly reduce the postharvest fish loss, as high temperature is one of the main factors of PHFL (Mandal et al. 2025; Prodhan et al. 2022). Proper and sufficient capacity for storage and availability of cold storage and warehouse facilities are considerably significant for reducing wastage and maintaining the agricultural product quality (Accorsi et al. 2017; Sharma and Singh 2011; Narula 2011; Negi and Anand 2015). The location where farmers sell their fish has a significant association with postharvest losses. The study found that farmers who sold their fish to their neighbors experienced a lower proportion of postharvest loss by 0.02 compared to those selling at other locations such as village markets, upazila markets, and district markets. This reduction in loss is attributed to the shorter storage time required when selling to neighbors, which helps minimize fish spoilage (Ejeta et al. 2024). Mandal et al. (2024) also emphasized the need to sell fish locally for reduced fish losses. The mode of transportation used by farmers for the movement of fish after harvesting to selling also influenced fish spoilage. Those who used trucks or pickups for the movement of fish experienced a reduction in the proportion of income loss by 0.09 due to postharvest spoilage of fish, which could contribute to their food security.

Distance from the market had a positive and significant relationship with fish losses. An increase in the distance of the village market by 1 km results in a 19% increase in fish postharvest loss, which results in a higher proportion of income loss by the fish farmers. Abelti and Teka (2024) stated that a longer distance from the market exacerbates fish spoilage, which is due to prolonged exposure and inadequate preservation practices, poor handling techniques, which also align with the findings of Assefa et al. (2018) and Agbebi et al. (2021). Farmers having transport facilities at their location experienced 9% more proportion of income than those with no such facilities. Having a transportation facility at the farmers' location provides them easy linkage to the market, which gives them the opportunity to sell their product at a fair price (Abdulraheem et al. 2021).

The estimates of the OLS model and the fraction probit model fitted for the determinants of postharvest loss at the traders' level are represented in Table 8, which is quite similar to the previous table. Like the farmers' level, the positive and significant determinants of fish postharvest loss at the traders' level were the age of the traders from 36 to 45 years, 46 to 55 years, and 56 to more than 65 years, service holder traders, transporting fish by bus and other modes, and the distance of the village market. An increase in the distance of the village market by 1 km would increase the fish postharvest loss of traders by 9%, which is similar to the findings of Assefa et al. (2018). Whereas traders having a secondary school certificate, 6–10 years of engagement, and more than 11 years of engagement in fish selling, training on fish preservation, traders' perception about the cooling method, perception about iced fish, and transportation facility at the farmers' location were significant negative determinants of fish postharvest loss. On the other hand, the fraction probit regression model shows that all the significant variables of OLS regression, along with traders' use of refrigerated vans as a mode of transportation and traders' education level of HSC, were significant determinants of fish postharvest loss.

**TABLE 8:** Determinants of postharvest losses at trader's level: Regression results.

Variables	OLS regression		Fraction probit regression			
	Total loss		Fraction of loss		Marginal effects	
	Coef.	Robust SE	Coef.	Robust SE	d*y*/d*x*	Robust SE
*Age category*						
26–35	1.19	1.01	0.06	0.04	0.01*	0.01
36–45	3.03**	1.30	0.14***	0.05	0.03***	0.01
46–55	4.81**	2.02	0.20***	0.07	0.04***	0.01
56–65+	6.87***	1.96	0.29***	0.07	0.06***	0.01
*Education category*						
High school	−0.18	0.75	0.00	0.03	0.00	0.01
SSC	−2.53**	1.11	−0.11**	0.05	−0.02***	0.01
HSC	−1.10	0.83	−0.10***	0.03	−0.02***	0.01
*Occupation*						
Business	−0.66	0.72	−0.04	0.03	−0.01	0.01
Service holder	3.49**	1.49	0.12***	0.04	0.03***	0.01
*Years of engagement in fish selling*						
6–10	−2.39***	0.78	−0.11***	0.03	−0.02***	0.01
11+	−2.92**	1.34	−0.13***	0.04	−0.03***	0.01
Training on fish preservation	−2.65***	0.97	−0.13***	0.04	−0.03***	0.01
Engagement with fish seller association	0.34	0.75	0.00	0.03	0.00	0.01
*Where do you mostly like to sell fish*						
Upazila market	−0.19	0.87	0.01	0.03	0.00	0.01
Dist. market	1.13	0.79	0.07**	0.03	0.01**	0.01
*Mode of transportation*						
Refrigerated van	−1.09	1.70	−0.13*	0.07	−0.02*	0.01
Truck and pickup	−0.08	0.78	−0.02	0.03	0.00	0.01
Bus and others	1.53*	0.97	0.06*	0.03	0.01*	0.01
Perception about cooling method	−2.04*	1.10	−0.08**	0.04	−0.02**	0.01
Perception about iced fish	−1.87**	0.82	−0.07**	0.03	−0.01**	0.01
Use of preservatives	−1.36**	0.64	−0.08***	0.02	−0.02***	0.00
Cooling facilities	0.09	0.67	−0.01	0.03	0.00	0.01
How far is the village market	0.09**	0.04	0.00***	0.00	0.00***	0.00
Transportation facility in farmers location	−2.14***	0.76	−0.09***	0.03	−0.02***	0.01
Infrastructure facility in farmers location	−0.47	0.74	−0.03	0.03	−0.01	0.01
Constant	15.20***	2.26	−1.06***	0.08		
Number of observations	95		95			
*F* (26, 37)/Wald *χ* ^2^ (27)	18.95***		827.64***			
*R*‐squared/pseudo *R* ^2^	0.85		0.03			

*Note:* ∗, ∗∗, ∗∗∗ indicate significance at 10%, 5%, and 1% level, respectively.

### 4.7 Major Problems in Fish Cultivation and Selling

Table 9 represents the major problems faced by the fish farmers and sellers during fish cultivation and fish selling. Lack of capital was the most prominent problem faced by the farmers, and it was also reported by 33% of sellers, whereas the major problem of sellers was lack of a cold storage system, and it was also reported by 54% of farmers. They faced a lack of a proper transportation system, political problems, and other issues.

**TABLE 9:** Major problems in fish cultivation and selling business.

Problems	Farmers (*N* = 65)	%	Sellers (*N* = 95)	%
Lack of capital	38	58.46	31	32.63
Lack of proper transportation system	11	16.92	12	12.63
Lack of cold storage system	35	53.85	71	74.74
Political unrest	—	—	7	7.37
Others' problem	20	30.77	10	10.53

*Note:* Cumulative sum of the percentage is not always 100% since multiple counts are accounted.

### 4.8 VC Actor's Recommendations/Suggestions to Reduce Postharvest Losses

Fish value chain actors were asked to suggest ways for the reduction of postharvest losses presented in Table 10, where 47% farmers, 97% sellers, and 16% consumers suggested developing the icing facility or storage facility. Improvement of water quality was the recommendation of 15% farmers and 6% sellers, whereas 20% farmers responded to ensure proper transportation, and only a few sellers responded to developing proper sanitation, providing training on proper handling, postharvest loss reduction, and development of infrastructure. Where the majority of consumers had no suggestions regarding the postharvest loss reduction.

**TABLE 10:** VC actor's suggestions to reduce fish postharvest losses.

Suggestions to reduce postharvest losses	Farmers (*N* = 65)	%	Sellers (*N* = 95)	%	Consumer (*N* = 60)	%
Develop icing/storage facility	31	47.69	92	96.84	10	16.13
Improve water quality	10	15.38	6	6.32	—	—
Develop proper sanitation	—	—	8	8.42	—	—
Training on proper handling and postharvest loss reduction	—	—	9	9.47	6	9.68
Develop infrastructure	—	—	6	6.35	—	—
Proper transportation	13	20	—	—	2	3.22
No idea	22	33.84	—	—	43	69.35

*Note:* Cumulative sum of the percentage is not always 100% since multiple counts are accounted.

## 5 Postharvest Fish Losses and Food Security Implications

The findings of this study indicate an average total postharvest loss (PHL) of approximately 10% at the farmer level and 12% at the trader level in the small‐scale fish value chain in the study area of Bangladesh, which has important implications for food security. These losses, which include physical, quality, and market‐driven decreases, directly reduce the quantity of fish accessible for consumption; therefore, undermining the availability pillar of food security (FAO 2010; Tesfay and Teferi 2017). Given that fish is a primary source of animal protein in Bangladesh (DoF 2019b), such losses can worsen nutritional deficiencies, particularly among vulnerable populations who rely on fish for vital micronutrients (Thilsted et al. 2016). The higher losses seen at the trader level suggest inefficiencies across the value chain, further reducing the net fish supply reaching consumers.

The perceived causes of PHL, elevated temperature/adverse weather conditions, lack of storage facilities, and spoilage rank high among farmers and sellers, highlighting major infrastructural and environmental challenges. These factors not only contribute to physical and qualitative losses but also impact the stability of fish supply and income for those dependent on the fisheries sector. Unfavorable weather conditions, likely exacerbated by climate change, can lead to unpredictable gluts or scarcities, affecting market prices and, consequently, the economic accessibility of fish for consumers and income stability for producers and traders (Chauhan et al. 2021). The reported lack of storage facilities, particularly cold storage, as highlighted by 54% of farmers and 71% of sellers as a major problem, is a critical bottleneck. Without adequate cold chain infrastructure, the shelf‐life of highly perishable fish is drastically reduced, leading to forced sales at lower prices (market loss) or outright spoilage (physical and quality loss), a concern also noted by Accorsi et al. (2017) and Narula (2011) in the context of perishable agricultural products. Poor fish handling practices, identified as the primary cause of PHL by consumers and fourth by farmers and sellers, directly affect fish quality and safety, impacting the utilization aspect of food security. Suboptimal handling can lead to microbial contamination and rapid deterioration, reducing the nutritional value and potentially posing health risks to consumers (Getu et al. 2015). Although the study shows a good general perception of cooling methods, the finding that only a small percentage of farmers (3%) and sellers (7%) used “excellent” quality ice suggests a gap between awareness and optimal practice, potentially due to cost or availability constraints. This aligns with findings by Mandal et al. (2025), who emphasized that proper cooling could significantly reduce PHL.

The socio‐demographic and operational factors identified as determinants of PHL offer pathways for targeted interventions. The finding that younger, more educated, experienced, and trained farmers and traders incur significantly lower losses underscores the importance of human capital development. Higher education likely enhances the ability to adopt improved technologies and practices (Acharjee et al. 2021; Chen et al. 2018; Abiodun and Cookey 2023), whereas experience provides practical knowledge in handling and marketing (Agbebi et al. 2021; Maulu et al. 2020). Training in fish preservation, as shown in this study and supported by Adelaja et al. (2018) and Ejeta et al. (2024), directly equips actors with skills to mitigate losses. Membership in associations, found to reduce losses for farmers, likely facilitates access to information, resources, and collective bargaining power (Abelti and Teka 2024; Ejeta et al. 2024), enhancing their resilience and food security.

Conversely, older individuals experiencing higher losses might indicate a reluctance or reduced capacity to adopt new methods (Langyintuo and Mungoma 2008; Malit et al. 2022), pointing to a need for appropriate age extension services. The increased losses associated with selling in distant markets and longer transportation distances, especially without adequate facilities like refrigerated vans (Wibowo et al. 2017), highlight the critical role of market proximity and efficient coordination (Assefa et al. 2018; Agbebi et al. 2021). When fishers must travel longer distances, especially with poor transportation infrastructure, the exposure time increases, leading to higher spoilage, directly impacting their income and the availability of quality fish in remote consumer markets. This reinforces the need for investment in rural infrastructure, including roads and market facilities, as suggested by Abdulraheem et al. (2021).

The finding that service holders (part‐time fish farmers/traders) incur higher losses suggests that divided attention may lead to suboptimal management of perishable fish products. This contrasts with those solely reliant on fishing, who, as Mandal et al. (2024) suggest, are more likely to prioritize best handling practices. This has implications for livelihood diversification strategies, suggesting that support for part‐time actors should include specific training on efficient postharvest management. The economic impact of these losses on fish farmers and traders is substantial. The average losses of 10%–12% represent a direct reduction in income, which can push vulnerable households further into poverty and food insecurity (Iruo et al. 2018). When farmers and traders are forced to sell spoiled or nearly spoiled fish at lower prices, or discard them altogether, their economic returns diminish, affecting their ability to purchase other food items and invest in improved fishing or preservation technologies, creating a vicious cycle of loss and food insecurity. The lack of capital, cited as a major problem by 58% of farmers and 33% of sellers, further constrains their ability to invest in loss‐reducing measures such as better equipment or storage.

The suggestions provided by the value chain actors themselves, particularly the overwhelming demand for improved icing/storage facilities (47% farmers, 97% sellers), underscore the perceived importance of cold chain development. Addressing these needs, along with providing training on proper handling and improving transportation, as also suggested, could significantly contribute to reducing PHL. Such reductions would not only increase the net availability of fish but also enhance the incomes of fishers and traders, thereby improving their own food and nutrition security and contributing to the overall food security of the region (Ward and Signa 2014). The implications extend beyond mere quantitative availability; improved quality and safety of fish would lead to better nutritional outcomes for consumers (Abbas et al. 2024). Therefore, addressing PHL in the small‐scale fisheries sector of Bangladesh is not just an economic imperative but a crucial step towards achieving broader food security and sustainable development goals.

## 6 Conclusion and Policy Implications

Reducing postharvest loss throughout commodity value chains is an important pathway to food and nutrition security in many developing countries including Bangladesh. Significant quantities of food crops are physically lost at different stages of the chain as food commodities move along their often long, complex and dynamic value chains. Lack of accurate measurement, understanding of the stages where losses are significant, associated factors within the postharvest value chains and value chain actors' perception remain major challenges to operationalizing postharvest loss mitigation strategies. Using OLS and fractional probit models, the study determines the main factors that contribute to postharvest losses among fish farmers and traders. Higher losses are largely caused by factors like age, market location, and transportation distance; older farmers and traders have more difficulties because of their lower capacity for adaptation. Farmers who sell in far‐off marketplaces lose more money, and the issue is made worse by poor storage and transportation conditions. On the other hand, knowledge of preservation techniques, training, experience, and education all successfully lower losses. Better loss mitigation is shown by farmers and traders with more knowledge, more experience, and cooling method training. Policy initiatives should prioritize youth participation in fish trade, better education and training initiatives, updated transportation and storage infrastructure, and strengthened market connections in order to lower postharvest losses. Strengthening infrastructure and access to preservation technologies (e.g., drying technology, see Islam et al. 2025) is crucial for minimizing fish postharvest losses and improving efficiency in the fish sector in Bangladesh.

## Author Contributions

**Md Jakiul Islam:** conceptualization, investigation, funding acquisition, writing – original draft, writing – review and editing, validation, methodology, project administration, resources, supervision. **Ripa Das:** investigation, data curation, validation, writing – review and editing. **Shahir Chowdhury:** investigation, writing – review and editing, validation, data curation. **Tanni Sarkar:** investigation, writing – review and editing, validation, data curation. **Habiba Khan Ilham:** investigation, validation, visualization, data curation. **Abu Hayat Md. Saiful Islam:** conceptualization, investigation, writing – original draft, methodology, validation, visualization, writing – review and editing, software, formal analysis, data curation, supervision, resources, project administration. **Esrat Jahan:** investigation, methodology, validation, visualization, writing – review and editing, data curation.

## Funding

The authors would like to gratefully acknowledge the Sylhet Agricultural University Research System (SAURES) for its financial support (SAURES‐UGC‐22‐23‐77). The corresponding author also acknowledges financial support as a part of the Feed the Future Food Systems for Nutrition Innovation Lab (Cooperative Agreement # 7200AA21LE00001 with Tufts University).

## Conflicts of Interest

The authors declare no conflicts of interest.

## Data Availability

The data that support the findings of this study are available on request from the corresponding author. The data are not publicly available due to privacy or ethical restrictions.

## Appendix

**TABLE A1:** Multicollinearity test of independent variables used in regression analysis: Variance inflation factor (VIF) results.

Variables	Farmers		Sellers	
	VIF	1/VIF	VIF	1/VIF
*Age category*				
26–35			3.96	0.25
36–45	4.87	0.21	5.55	0.18
46–55	4.86	0.21	4.18	0.24
56–65	4.94	0.20	2.42	0.41
*Education category*				
High school	2.39	0.42	2.13	0.47
SSC	2.54	0.39	1.41	0.71
HSC	1.59	0.63	2.19	0.46
Diploma	2.49	0.40		
Bachelor	1.53	0.65		
*Occupation*				
Business	3.39	0.30	1.90	0.53
Service holder	1.75	0.57	1.69	0.59
*Engagement in fish cultivation (years)*				
6–10	3.93	0.25	2.32	0.43
11+	3.39	0.30	3.21	0.31
Training	2.34	0.43	1.61	0.62
Engagement with association	3.90	0.26	1.94	0.52
Contact with fishing personnel	2.91	0.34		
Perception about cooling method	3.10	0.32	2.43	0.41
Perception about iced fish	4.19	0.24	1.42	0.70
Use of preservatives in fish selling			2.13	0.47
Cooling facilities			2.53	0.40
*Places of fish sale*				
Neighbor	1.79	0.56		
Village market	2.32	0.43		
Upazila market	2.61	0.38	4.61	0.22
District market	2.76	0.36	3.86	0.26
*Mode of transportation*				
Refrigerated van	2.54	0.39	2.25	0.44
Truck or pickup	5.14	0.19	3.24	0.31
Bus or others	7.16	0.14	3.34	0.30
How far is the village market	2.11	0.47	3.00	0.33
Transportation facility in farmers location	3.81	0.26	2.03	0.49
Infrastructure facility in farmers location	4.06	0.25	2.38	0.42
Mean VIF	3.27		2.71	
